# Draft Genome Sequence of *Streptomyces* sp. AVP053U2 Isolated from *Styela clava*, a Tunicate Collected in Long Island Sound

**DOI:** 10.1128/genomeA.00874-16

**Published:** 2016-10-13

**Authors:** James A. deMayo, Kendra R. Maas, Jonathan L. Klassen, Marcy J. Balunas

**Affiliations:** a Division of Medicinal Chemistry, Department of Pharmaceutical Sciences, University of Connecticut, Storrs, Connecticut, USA; b Microbial Analysis, Resources, and Services, University of Connecticut, Storrs, Connecticut, USA; c Department of Molecular and Cell Biology, University of Connecticut, Storrs, Connecticut, USA

## Abstract

*Streptomyces* sp. AVP053U2 is a marine bacterium isolated from *Styela clava*, a tunicate collected in Long Island Sound. Here, we report a draft genome for this bacterium, which was found to contain a high capacity for secondary metabolite production based on analysis and identification of numerous biosynthetic gene clusters.

## GENOME ANNOUNCEMENT

Actinomycetes from a variety of marine environments have demonstrated a strong potential for the production of biologically relevant secondary metabolites. Strains from the *Streptomyces* genus are known to be prolific producers of antibiotics and other biologically relevant secondary metabolites (1), with many of these molecules incorporating moieties derived from nonribosomal peptide synthetase (NRPS) and/or polyketide synthase (PKS) biosynthetic gene clusters (2). The incorporation of terpenoid/isoprenoid moieties is far less common in actinomycetes (3). Here, we report the genome sequence of a new strain of marine-derived *Streptomyces* sp. AVP053U2 that contains a biosynthesis gene cluster responsible for the production of teleocidin B, a hybrid isoprenoid compound related to teleocidin A1, a toxic tumor promoter acting via PKC activation (4, 5). *Streptomyces* sp. AVP053U2 was isolated from homogenate of *Styela clava*, a tunicate collected from Avery Point, CT, USA (41°18′58.842″N, 72°3′38.682″W) in Long Island Sound.

*Streptomyces* sp. AVP053U2 was isolated and cultivated on Difco ISP-4 agar incubated at room temperature until sporulation occurred. DNA was extracted from these cultures using the Promega Wizard genomic DNA purification kit following the manufacturer’s protocol, quantified using a Qubit bioanalyzer, and fragmented to 550 bp using a Covaris sonicator. The Illumina TruSeq Nano DNA library preparation kit was used to attach adapter sequences and size select, according to the manufacturer’s protocol. Libraries were validated using an Agilent Bioanalyzer high-sensitivity chip to calculate mean insert length. Libraries were sequenced on the Illumina MiSeq using a v2 kit (2 × 250 bp) to 153× coverage, assembled with the A5-miseq pipeline version 20140113 (6), checked for contamination with the Blobology pipeline (7), and annotated with Prokka version 1.10 (8). Bidirectional average nucleotide identity (ANI) between *Streptomyces* sp. AVP053U2 and its closest related genome, *Streptomyces* sp. TP-A0873 (NBRC 110035, accession no. BBNN00000000), was 99.68% (9).

The AVP053U2 genome assembly comprises 7,759,417 nucleotides in 183 scaffolds (*N*_50_, 137,942 bp; 71.9% G+C content). Examination using antiSMASH version 3.0.4 (antibiotics and Secondary Metabolite Analysis Shell) (10) identified 46 putative secondary metabolite biosynthetic gene clusters. Six shared 100% identity with previously reported clusters for teleocidin B, isorenieratene, albaflavenone, antimycin, γ-butyrolactone, and ectoine biosynthesis. The remaining clusters included 11 NRPSs, seven type-1 PKSs; six terpene; three lantipeptide; two NRPS/type-1 PKSs; two siderophores; one each of amgylccycl/NRPS, type-1 PKS/transAT PKS/NRPS, terpene/type-2 PKS, indole/NRPS, melanin, NRPS/lantipeptide, butyrolactone/type-1 PKS, butyrolactone, nucleoside, ectoine, bacteriocin, NRPS/ladderane-arylpolyene, and type-3 PKS; and two unidentified clusters. Fewer than half of the annotated gene clusters shared high homology to those from antiSMASH analysis of *Streptomyces* sp*.* TP-A0873, with only 20 clusters shared between the two genomes.

This analysis indicates a strong potential for secondary metabolite production by *Streptomyces* sp. AVP053U2. While the AVP053U2 genome includes a biosynthetic gene cluster with high homology to the teleocidin B gene cluster, the AVP053U2 cluster lacks the final methyltransferase necessary to convert teleocidin A1 to teleocidin B (11). Analysis of AVP053U2 secondary metabolites may provide further insight into the production of teleocidin derivatives.

### Accession number(s).

This whole-genome shotgun project has been deposited in DDBJ/ENA/GenBank under the accession number LMTQ00000000. The version described in this paper is the second version, LMTQ02000000.
